# Changes in biochemical parameters by gender and time: Effect of short-term vegan diet adherence

**DOI:** 10.1371/journal.pone.0237065

**Published:** 2020-08-12

**Authors:** Tariku Sisay, Tesfaye Tolessa, Wondyefraw Mekonen

**Affiliations:** Department of Physiology, School of Medicine, College of Health Sciences, Addis Ababa University, Addis Ababa, Ethiopia; University of Hawai’i at Manoa College of Tropical Agriculture and Human Resources, UNITED STATES

## Abstract

**Background:**

Vegetarian diets adapted for various reasons that may include religious, ethical, and health considerations have reasonable health benefits including weight loss, and favorable metabolic changes. However, studies that assessed health benefits associated with vegan diet practices during the Ethiopian Orthodox Christian (EOC) Lenten fasting remains limited. This study has, therefore, assessed how short-term vegan diet associated with metabolic traits, including weight, body mass index (BMI), circumference, blood pressure, total cholesterol (TC), triglycerides (TG), high-density lipoprotein cholesterol (HDL-C), and low-density lipoprotein cholesterol (LDL-C), through longitudinal cross-sectional study design.

**Methods:**

Seventy-five subjects (34 females and 41 males) with a mean age of [+SD] 27.3 + 5.8 years (range, 18 and 35) took part in the study. The study followed three assessment sessions: at baseline, during the Lenten (week 7), and 7 weeks after the end of the Lenten (week 14). An automatic chemistry analyzer (Mindray, BE-2000, China) used for lipid profile analysis. We used paired sample t-test in pre and post-performance and repeated measures ANOVA with Bonferroni post hoc adjustment between time points. The statistical significance was set at p < 0.05.

**Results:**

The EOC fasting with vegan diet induced significantly lower blood pressure, weight, BMI, TC, HDL-C, LDL-C, and TC: HDL-C ratios, during Lenten (that is vegan diet consumption), but a regain noted in these parameters 7-weeks after Lenten (that is omnivore diet). On gender differences, vegan diet associated with significantly lower blood pressure, TC, and LDL-C in females compared with age-matched male counterparts. Some methodological limitations of this study are discussed with particular reference to lack of a randomized control group and self-reported data that limit this study in establishing a causal relationship through observed associations.

**Conclusions:**

Vegan diet consumption even for short period corroborate ideal metabolic traits, with more favorable changes noted in women than age-matched men counterparts. These findings might help to define vegetarian diets as part of religious fasting (beyond its spiritual goals) as a non-pharmacological prescription in different populations, and our findings add to growing evidence in these subjects.

## Introduction

Individuals around the world follow vegetarian or plant-based diets (PBDs), but in most countries. vegetarians comprise only a small proportion of the population. While it is difficult to separate the good effects of a healthy diet on cardiovascular disease (CVD) from those of regular exercise, nonsmoking and maintaining a BMI < 25 kg/m^2^, earlier data showed that chronic diseases are favorably altered by the regular consumption of PBDs with or without the low amount of non-fat dairy products [1, 2]. The serum concentration of HDL-cholesterol, LDL-cholesterol, TG and the ratio of HDL-C to TC tend to be lower in PBDs and are often used to predict the risk of having CVD [4, 5].

Religious fasting (beyond its spiritual goals) could offer an opportunity to follow vegetarian diets and this may in turn lead to reasonable health benefits including weight loss, and favorable metabolic changes [3–6]. Fasting, the willful partial or total abstention from restricted foods in many religions [2, 6]. Research studies in Seventh-day Adventists [7], Greek Orthodox Church [8], and Egyptian Copts [9] have shown that religious fasting could play favorable health benefits. Different from the EOC-fasting episodes, the Greek Orthodox Church [10] and other studies [11, 12] allow the faithful to consume seafood and fishes. The Ethiopian Orthodox Church specifies dietary limits and fasting for the total number of 250 days each year, of which about 180 days are compulsory for all. These fasting practices are performed on every Wednesdays and Fridays, and the entire Lenten season [13]. To the EOC, Lenten is a test of one’s Christianity with greater rigor than any other fasting that encompasses 56 days. During the lent fasting most of the EOC considered as “vegans”, since meat, eggs, dairy, and honey are restricted. Other lifestyle behaviors (for example., alcohol use, and smoking, sexual) also restricted specifically during Lent [13]. After the Lenten (Easter) to Seventh Sunday, a faithful may eat and drink what they like including every Wednesday and Friday.

Premenopausal females are shown with ideal metabolic traits than age-matched males. This could be the difference in attitudes and notions of PBDs choice between men and women. Several studies have highlighted that men consume more non-vegetarian diets, and various high starch foods such as potatoes and bread. Women, however, consume various vegetarian diet (fruits, vegetables, cereal, milk, and whole-grain products) [14, 15]. It is also possible that PBDs including higher amounts of refined grains, potatoes or fries, sweets, and sweetened drinks, were linked to more weight gain over time. To our knowledge, studies that assessed health benefits associated with a vegan diet practices during the EOC-fasting episodes by gender remains limited. This study has, therefore, assessed favorable changes in consuming vegan diet even for short period on changes of biochemical and anthropometric parameters by gender on a group of EOC fasting subjects.

## Materials and methods

### Ethical statement

This study conducted following the guidelines laid down in the Declaration of Helsinki and approved by the Ethics Committee of School of Medicine, College of Health Sciences, Addis Ababa University (Anat/phy 286/2017). Participants provided written consent to participate, with an explanation of procedures, and risks and benefits in the study. Privacy issues for all measurements, including separate facilities or areas in a room or times for women and men kept. The laboratory staff was blinded to the participant’s identity and the samples coded with a unique identifier-not named.

### Subjects

Seventy-five subjects (34 females and 41 males) with a mean age of [+SD] 27.3 + 5.8 years (range, 18–35) took part in the study. All participants were members of Ethiopian Orthodox Church, who practice religious Lenten fasting by taking a vegan diet. Recruiting was carried out through personal contacts, advertisements at churches and universities, where all participants were residents of the capital city, Addis Ababa (~4 million citizens).

### Eligibility

Each participant told to appear immediately in the laboratory for each sessions. All subjects were confirmed to be EOC in that they had followed religious fasting by restring any animal-traced foods throughout the Lenten fasting (eight weeks). We excluded smokers, alcoholics, pregnant women, and those who suffered from any chronic diseases or on any medication.

### Assessments and measures

We used three assessment sessions: start of the Lenten (baseline), after the Lenten (week 7), and 7 weeks after the end of the Lenten (week 14). Metabolic traits, including height (m), weight (kg), BMI (kg/m2), circumference, blood pressure, total cholesterol triglycerides (TG), high-density lipoprotein cholesterol (HDL-C), and low-density lipoprotein cholesterol (LDL-C), assessed through longitudinal cross-sectional study design. All measurements and the completion of questionnaires have done in the morning hours between 08.00 a.m. and 10.00 a.m.

#### Anthropometric variables

Bodyweight (kg) without shoes and with light clothing using a beam balance (Seca, GmbH, Germany) taken and rounded to the nearest 0.5 kg. Height at standing position with heads backs and buttocks vertically aligned to the height gauge taken and rounded to the nearest 0.5 cm. The standard tape used to measure waist circumference (WC) at the narrowest point between the highest point of Iliac crest and the lower ribs margin [16]. Three consecutive measurements of height, weight, and WC recorded for each subject and averaged for analysis.

Body mass index (BMI), the ratio of weight in kilograms to height in square meters [Weight (kg)]/[height (m)]2, calculated to the nearest decimal place.

#### Blood pressure

An automatic monitor (Omron HEM-780, Japan) used for blood pressure (BP). Before taking BP readings, all subjects rested for five minutes in an air-conditioned environment. Three BP measurements were taken on the right arm using a proper cuff size with short intervals between readings, and the average of the last two readings used for analysis.

#### Biochemical analysis

Blood was collected after fasting confirmed for at least 12 hours and carried out standard procedures for blood samples. Five milliliters of venous blood samples drawn into serum separator tubes by a qualified and skilled professional. Serum TC, TAG, and HDL-C were assayed enzymatically to standard laboratory procedures using an automatic chemistry analyzer (Mindray, BE-2000, China). LDL-C estimated using Friedewald’s equation [17]. All tests analyzed at the Diagnostic Laboratory of Black Lion School of Medicine, Addis Ababa University.

#### Dietary intakes

Habitual dietary intakes were assessed through a self-administered food frequency questionnaire (FFQ) that is modified to reflect the EOC Lenten fasting diets in its format. The questionnaire was first written in English, then translated to local language (Amharic) and back to English for its consistency. Pretest of the questionnaire was conducted in 7 volunteer subjects (~10% study sample size) for validation of FFQ two weeks before data collection and some adjustment added. For each item, the participants asked to select one frequency categories that best characterized their eating habits over the three assessment sessions: ‘daily’, ‘weekly’, ‘monthly’, and ‘never’., The food items were categorized to: bread, cereals, potatoes, vegetables, pulses, fruit, eggs, cheese, milk and yogurt, fats, pastries, meat, and fish.

#### Physical activity

Participants needed to be healthy and sedentary. Lifestyle physical activity was assessed using the International Physical Activity Questionnaire (IPAQ). The questions ask about the time spent in the last 7 days including in vigorous physical activities like heavy lifting, digging, aerobics, or fast bicycling; moderate activities that take moderate physical effort and make an individual breathe harder than normal; walking to travel from place to place, and any other walking that an individual has done solely for recreation, sport, exercise, or leisure; and sitting at a desk, visiting friends, reading, or sitting or lying down to watch television. Participants with ≤ 60 minutes of planned physical activity a week is considered as physically idle.

## Statistics analysis

Statistical analyses were performed using the Statistical Package for Social Sciences (SPSS) (version 21.0; IBM, Armonk). After complete entry of all the data, a soft copy was checked with its hard copy to see the consistency. Data entry was programmed in such a way that outlier entries were not accepted. Paired sample t-test was used to assess the differences in pre and post-performance tests between sex and repeated measures ANOVA and Bonferroni post hoc test were used to determine differences for blood samplings and anthropometric parameters between time points. GPower Version 3.1.9.2 was employed as a tool for the study design. Cohen’s d (standardized mean differences) was calculated for each dependent variable from the raw data, and effect size (0.5); power (1-β) of the study (0.95) and α-error probability (0.05) was used. Data are presented as mean ± SD. The statistical significance was set at p < 0.05.

## Results

### Participant characteristics

From the 87 participants recruited at first, seven participants excluded based on the inclusion and/or exclusion criteria. Eighty-one subjects were approached and provided with the data, checklists, and six subjects did not complete the study due to the lack of willingness or absence. Seventy-five subjects (41 men and 34 women) aged between 18 and 35 years (mean age 27.3 [SD 5.8] years), took part in this study. All participants were nonsmokers, healthy, and sedentary.

### Measurements

Table 1 shows changes in anthropometric and biochemical parameters by gender and time. Overall, we noted lower values for DBP, SBP, Weight, BMI, TC, HDL-C, LDL-C, and TC: HDL-C ratios, during Lenten (i.e., vegan diet consumption), but a regain was observed in these parameters 7 weeks after the Lenten (i.e., omnivore diet). While participants have adhered to vegan diets, a significant lower SBP, DBP, TC, and LDL-C was observed in women compared with age-matched male counterparts than during a vegetarian diet consumption.

**Table 1:** Comparison of changes in anthropometric and biochemical profiles by gender, and time on a group of 75 Ethiopian Orthodox Christian fasting subjects. All values are expressed as mean ± SD.

	Male (n = 41)			Female (n = 34)				
Variables	Before Lenten mean (SD)	Lenten mean (SD)	After Lenten mean (SD)	Before Lenten mean (SD)	Lenten mean (SD)	After Lenten mean (SD)	P-value for sex	P-value for time
Age (yrs)	28.3 (4.8)	_	_	25.3 (5.8)	_	_	_	_
Height (m)	1.71 (0.8)	_	_	1.67 (0.6)	_	_	_	_
SBP (mmHg)	112.8 (10.8)	109 (11.9)	107 (10.1)	100.3 (13.1)	117.1(10.1)	103.6 (8.9)	**0.041**	**0.013**
DBP (mmHg)	83.7 (10.2)	80.9 (10.0)	81.5 (11.4)	84.2 (9.7)	81.2 (7.9)	82.8 (6.9)	0.023	**0.013**
Weight (kg)	70.5 (8.6)	69.7 (8.9)	68.1 (8.7)	68.9 (8.4)	65.3 (8.9)	65.2 (8.9)	0.061	**0.02**
WC (m)	90.9 (10.9)	86.2 (11.0)	84.5 (11.1	70.6 (7.1)	68.7 (7.2)	68 (7.9)	0.057	0.123
BMI (kg/m2)	27.1 (3.4)	25.8 (3.4)	26.7 (3.5)	27.2 (5.5)	27.0 (5.7)	27.2 (5.5)	0.35	**0.012**
TC (mg/dl)	151.7(30.9)	144.2 (31.3)	157.2(30.3)	137.1 (31.7)	130.1(35.5)	130 (30.3)	**0.041**	**0.031**
TAG (mg/dl)	92.8 (36.1)	89.5 (36.3)	91 (35.7)	77.9 (39.1)	76.4 (38.3)	76.9 (39.1)	0.21	0.317
HDL-C (mg/dl)	47.8 (7.9)	45.2 (8.6)	45.9 (9.3)	54.5 (8.6)	51.5 (8.5)	53.9 (9.1)	0.21	**0.042**
LDL-C (mg/dl)	94.7 (26.1)	91.3 (25.6)	93.8 (26.4)	90.3 (32.2)	87.4 (34.5)	89.7 (33.5)	**0.012**	**0.029**
TC: HDL-C	2.3 (0.9)	1.9 (10)	2.7 (1.1)	90.3 (32.2)	3.6 (0.8)	3.7 (3.9)	0.137	**0.021**
LDL-C: HDL-C	2.5 (0.7)	2.3 (0.8)	2.4 (0.9)	2.3 (1.1)	2.1 (0.8)	2.1 (0.9)	0.105	0.206

WC: waist circumference; DBP: diastolic blood pressure; SBP: systolic blood pressure; WHR: waist-to-hip ratio; TC; total cholesterol, TG; triglyceride, HDL-C; high density Lipoprotein-C, LDL; low density lipoprotein.

P_**1**_: independent t-test was used to assess the differences between males and females at week 7 and week 14; **P**_**2**_: repeated measures ANOVA and Bonferroni post hoc test was used to determine differences between three time points.

**Fig 1:**
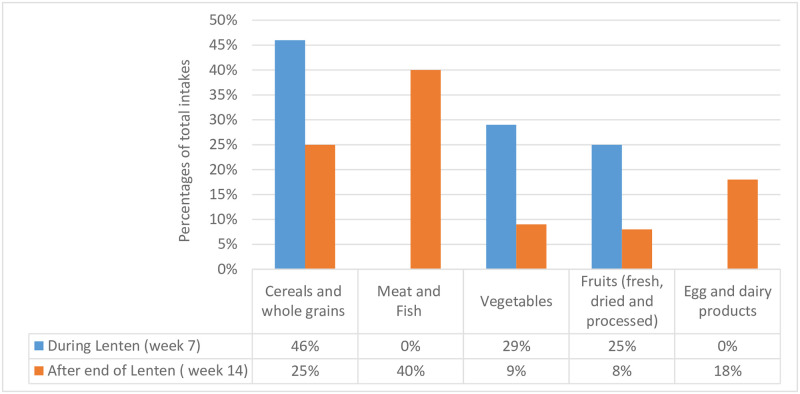
Percentages of animal and plant origin food intakes during and after the Lenten fasting.

As shown in Fig 1, the participants were restricted from dairy products and eggs including meat during the Lent period. For seven weeks after Lent fasting (Easter), however, all participants eat what they like including every Wednesday and Friday.

## Discussion

This study compared health benefits associated with consuming vegan diet during EOC-fasting with meat containing omnivorous diet on changes of metabolic parameters. Both the omnivorous diet and the vegan diet were consumed ad-libitum without pre-specified calorie restrictions. During the omnivorous dietary intake, participants reported to have taken meat products mixed with plant diet at least five or more servings a week.

The vegan diets that consumed for the entire 7-weeks of the EOC Lenten were purely a plant based diets that involved whole grains, cereals, green leafy vegetables, legumes, peas, beans, fruits [13]. A local stew (wat) that scooped up with enjera (sour flatbread made with teff, a tiny, round grain) is the habitual foods of EOC during Lent. The particular stew is Shiro wat (made from chickpea, broad bean flour, mixed with garlic and onions, and made into a thick, just like a paste), miser wat, or red lentil stew and Salata (Ethiopian salad). The physical activity of all participants in this study was minimal and they upheld a sedentary lifestyle (≤60 minutes of structured or planned physical activity a week). Therefore, we believe the changes in the physical activity of the subjects had no impact on the gained results.

### Changes on anthropometric parameters

We noted that participants experienced a noteworthy decrease in body weight and BMI during vegan diet adherence (that is 7-weeks of Lenten fasting), but most individuals regained weight seven weeks after the end of the Lenten (that is omnivorous diet consumption). The present study is in line with those of studies conducted on Seventh-day Adventist [18, 19] and Greek Orthodox Christian [19, 20]. Another line of evidence showed that weight and BMI of vegetarians were significantly lower than that of omnivore men is studied in Buddhist monks [21, 22]. However, the obvious health benefits of PBDs patterns may relate to other lifestyle factors (for example, more physical activity, lower alcohol, and tobacco use) [23]. It is also possible that PBDs including higher amounts of refined grains, potatoes or fries, sweets, and sweetened drinks, were linked to more weight gain over time. Nevertheless, the weight loss associated by the vegans could be explained in part by the low-glycemic values and the abundance of viscous fiber in whole grain products and vegetables, in whole grains in vegan diets [24–26].

### Changes in blood pressure

Several evidences pointed out an important role of vegetarian diets in reducing blood pressure (BP) and age-adjusted prevalence of hypertension than in non-vegetarians [22, 27, 28]. Our finding adds to previous researches from our short-term vegan dietary adherence associated with significantly lower systolic and diastolic BP levels. This could be due to lower BMI rather than diet [29] or could be because of high in potassium and fibers that accounted for lower BP in vegetarians [21].

Earlier studies on gender differences in BP showed that men had higher 24-hour mean blood pressure than in premenopausal women [21, 30]. After menopause, however, blood pressure was similar for men and women [30]. Our findings marked a significantly lower BP in females than in males at similar ages. Although the mechanisms are not clear, a shred of significant evidence that sex hormones (e.g., androgens, such as testosterone), play an important role in gender-associated differences in BP regulation [21, 30]. It is worthy to note that averaging blood pressure over 24 hours would reduce gender differences [30].

### Changes on biochemical profiles

It is known that lipids being insoluble in water, are transported in the blood bound to specific proteins called Apolipoproteins. It should be emphasized to note lipoproteins measured in clinical practice include: chylomicrons, VLDLs, LDLs, and HDLs consist of varying amounts of TG, cholesterol, phospholipid, and protein leukotrienes [31]. In many situations, the concentrations of these lipids and/or lipoproteins are not in normal amounts in the human body.

Studies pointed out that a favorable lipid profile is more common among vegetarians than in non-vegetarians [32]. This may be true due to vegetarians; especially vegans may have more fiber intake than those of non-vegetarians [33]. Several cross-sectional studies on lipid profiles found that TC, LDL-C, and TG levels were lower in the vegetarians than in semi-vegetarians and omnivorous [34, 35].

Overall, we noted a significantly lower TC, HDL-C, LDL-C, and TC: HDL-C ratios after consumption of vegan diets as short as 7-weeks followed the EOC Lenten fasting. The effect of dietary patterns on the TG level has been inconsistent. Some studies have suggested that a vegetarian diet is associated with higher TG levels than with a non-vegetarian diet [35], while a few did not note remarkable changes [28].

Similarly, there have been mixed findings on HDL-C and TG concentrations in different vegetarians’ dietary patterns. For instance, some studies reported a significantly lower HDL-C in a vegetarian than in a non-vegetarian [24]. While few studies did not remark a significant difference in HDL-C between a vegetarian and a non-vegetarian diet groups [28, 36]. HDL-cholesterol is associated with cholesterol removal. High concentrations are worthwhile and inversely related to cardiovascular diseases [37]. However, lower HDL-C in vegetarians that could be due to low-fat diets is unlikely to present a risk for disease-related to cardiovascular [38].

On gender differences, our finding suggests that female participants experienced a significantly higher HDL-C, and lower TC and LDL-C levels compared with male participants during the consumption of a vegan diet than during vegetarian diet consumption. To the best effort of authors, however, studies that assessed vegetarian and/or a non-vegetarian diet with gender remains limited. Generally, premenopausal females are shown with higher HDL-C and lower LDL-C and TG than age-matched males [39, 40]. The mechanisms for such favorable lipid profiles in females maybe because of sex hormones, also hormone-independent effects of the sex-chromosomes in tissues, could induce throughout the body [41].

### Limitations and strength

The lack of a randomized control group and self-reported data limit this study in establishing a causal relationship through observed associations. Besides, the mean duration of daily fasting in this study was about 6 hours. Therefore, the findings of this study cannot be generalized to other settings that experience long hours with caloric control.

It should be noted that this study was intentionally emphasized to corroborate if significant changes on metabolic traits, including weight, BMI, circumference, blood pressure, and lipid profiles could be observed in as little as 7-weeks duration. Explaining these findings might help to define vegetarian diets as part of religious fasting (beyond its spiritual goals) as a non-pharmacological prescription in different populations, and our findings add to growing evidence in these subjects.

## Conclusion

Our findings remarked that vegan diet consumption as short as seven weeks corroborate ideal metabolic traits, including weight loss and lipid profiles with more remarkable changes observed in females than age-matched male counterparts. Consuming plant-based diets as part of religious fasting and/or daily life is beneficial to optimize cardiovascular risk factors. The purpose of this study, however, is to inform further research, and our data features novel findings that should now inform longer-term randomized controlled trials.
